# A systematic review of microgravity on reproductive systems: Implications for space biology and human health

**DOI:** 10.1016/j.isci.2026.114663

**Published:** 2026-01-12

**Authors:** Chun-Fan Lung, Hsuan-Hsuan Lu, Chen-Yen Chung, Pao-Tai Lin, Yi-Chiung Hsu

**Affiliations:** 1 Department of Biomedical Sciences and Engineering, National Central University, Taoyuan, Taiwan; 2 Department of Melanoma Medical Oncology, University of Texas (UT) MD Anderson Cancer Center, Houston, TX, USA; 3 iPreg Inc., New Taipei City, Taiwan; 4 Soochow University Department of Microbiology, Taipei, Taiwan; 5 Department of Electrical and Computer Engineering, Texas A&M University, College Station, TX 77843, USA; 6 Department of Materials Science and Engineering, Texas A&M University, College Station, TX 77843, USA; 7 Center for Remote Health Technologies and Systems, Texas A&M University, College Station, TX 77843, USA; 8 Center for Astronautical Physics and Engineering, National Central University, Taoyuan, Taiwan; 9 Department of Medical Research, Cathay General Hospital, Taipei, Taiwan; 10 Department of Space Science and Engineering, National Central University, Taoyuan, Taiwan

**Keywords:** reproductive medicine, space medicine

## Abstract

Microgravity poses risks to mammalian reproductive health by altering gametogenesis, fertilization, and embryonic development. This review summarizes evidence from spaceflight and ground-based microgravity analogs, together with radiation exposures, showing disruptions in spermatogenesis, oogenesis, and pre-implantation embryo development that depend on cell type, exposure duration, and experimental platform. Mechanistic studies indicate that microgravity and radiation can trigger oxidative stress, DNA damage, mitochondrial dysfunction, and epigenetic alterations, potentially reducing gamete quality and embryo viability. We also highlight extracellular vesicles as key mediators of gamete maturation, fertilization, and implantation; microgravity-related changes in EV biogenesis or cargo may further impair reproductive success. In addition, spaceflight-associated microbiome shifts—including emerging evidence on reproductive-tract microbiomes—may influence fertility via immune dysregulation and inflammation. Finally, we discuss countermeasures such as artificial gravity, dietary and antioxidant/radioprotective strategies, and reproductive technologies, and outline priorities for future research to safeguard reproductive health in space and advance reproductive medicine on Earth.

## Graphical abstract

**Figure:**
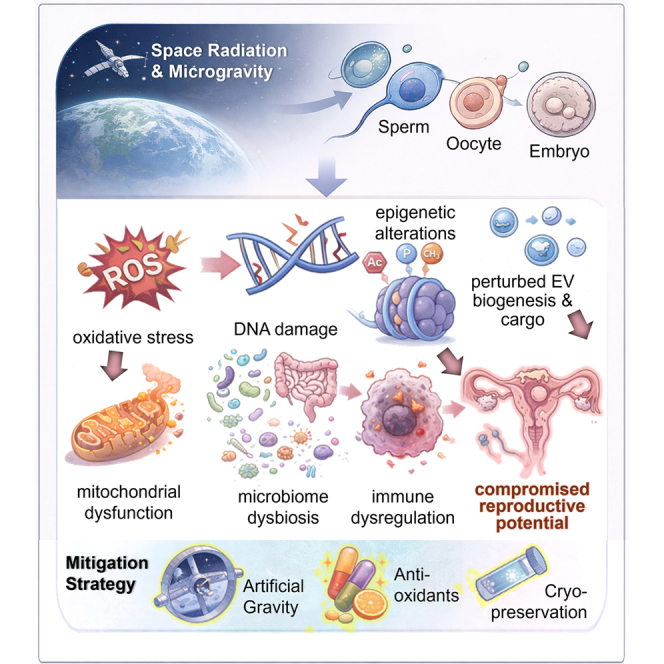

## Introduction

Human reproductive health is shaped by multiple stressors—including age, lifestyle,^1^ infection,^2^ occupational hazards, and environmental pollution^3^^,^^4^—that can impair gametogenesis, fertilization, and embryo development.^1^ Specifically, microgravity and space environments pose significant challenges to mammalian reproduction and embryonic development. Exposure to the unique conditions of space—characterized by microgravity and increased radiation—has been shown to induce a wide array of physiological changes in both male and female reproductive systems. Animal studies have suggested that microgravity and radiation can independently and synergistically disrupt reproductive functioning across various species, affecting processes such as spermatogenesis, oogenesis, and early embryonic development.^5^^,^^6^^,^^7^ These effects are described in detail later in this review, and such insights are vital for future long-duration missions and the goal of human colonization beyond Earth.

Although human pregnancy in space is currently contraindicated, understanding the biological processes underlying reproduction in space environments remains an important area of research. Key areas of concern include the effects of simulated or spaceflight microgravity on gynecological and obstetric parameters, male fertility, and gamete quality—all of which could compromise human reproductive health during extended space missions.^5^^,^^6^^,^^8^^,^^9^ For instance, studies have found that microgravity significantly decreases sperm motility and vitality, raising concerns about the feasibility of natural conception in space.^8^^,^^10^^,^^11^^,^^12^^,^^13^ Microgravity exposure has also been shown to induce apoptosis and DNA damage in spermatogenic cells, further contributing to reduced fertility potential.^14^

**Figure 1:**
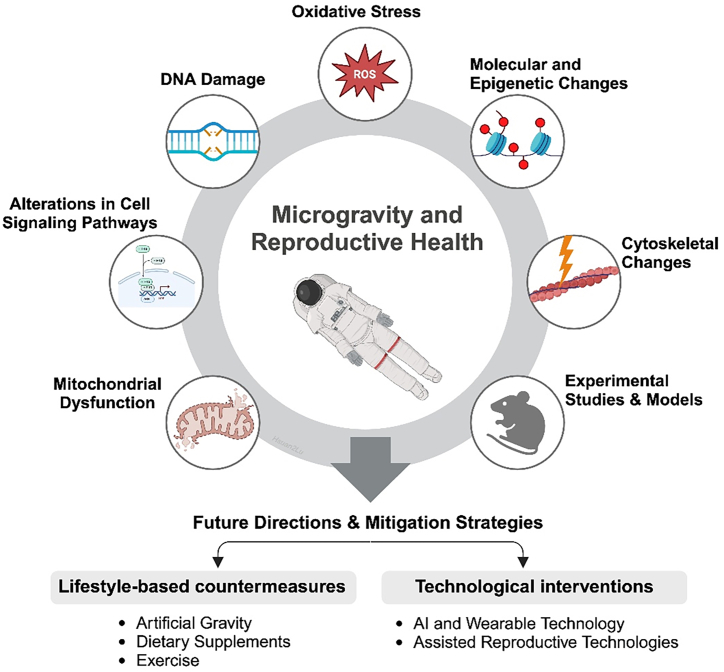
Impact of microgravity on reproductive health and mitigation strategies The circular diagram illustrates the major mechanisms through which microgravity affects reproductive health, including hormonal disruption, altered gene expression, and cellular changes. Contributing factors such as radiation, stress, and fluid redistribution are also depicted. The lower section outlines potential mitigation strategies and future research directions aimed at preserving reproductive health during space missions.

At the cellular level, microgravity induces significant changes, including alterations in the cytoskeleton, DNA integrity, oxidative stress, mitochondrial function, and epigenetic regulation (Figure 1). These responses have been observed across a range of systems, from germ cells and embryos to non-germ cell types such as fibroblasts, lymphoid, and stem cells. Specifically, genomic alterations driven by DNA damage and oxidative stress have been observed in human lymphoid and fibroblast cells under microgravity accompanied with radiation, with implications for reproductive health.^15^^,^^16^^,^^17^ Similarly, structural and functional changes in the cytoskeleton have also been noted in oocytes, embryos, and fibroblasts, which are crucial for proper cell division and stability, thereby affecting the overall viability of reproductive cells.^11^ Furthermore, extracellular vesicles (EVs), nanoscale, membrane-bound particles secreted by most cells, mediate intercellular communication integral to reproductive health. EVs comprise (i) exosomes—formed via endosomal multivesicular bodies and released upon fusion with the plasma membrane (ESCRT-dependent or ceramide-mediated)—and (ii) microvesicles—which bud directly from the plasma membrane; their use as umbrella term “EVs” is recommended by ISEV due to overlapping size/markers.^18^ EV cargo includes proteins, lipids, as well as nucleic acids (mRNA, miRNA, and other ncRNAs), which can reprogram recipient cells after uptake by endocytosis, membrane fusion, or receptor-mediated pathways; internalized EVs may be retained, degraded, or even re-released.^19^ In reproductive tissues and fluids, EVs from the oviduct, endometrium, and uterine fluid modulate gamete maturation, fertilization, and embryo implantation.^20^^,^^21^^,^^22^^,^^23^ Under microgravity conditions, the release and cargo composition shift of EVs indicate stress-responsive remodeling of paracrine signaling that could affect reproductive success.^24^ Clarifying EV biogenesis, cargo, and uptake under space conditions may therefore be essential for preserving gamete competence, fertilization, and embryo implantation during long-duration missions.

The astronaut microbiome is another important aspect that can influence reproductive health in space. Spaceflight conditions have been shown to cause significant changes in the microbiome, which can affect immune function, inflammation, and overall health.^25^^,^^26^ These changes can indirectly impact reproductive health by increasing the risk of infection and immune dysregulation. Maintaining a healthy microbiome during space missions is crucial for overall health, as changes in the microbiome can affect immune function and increase the risk of infections, which could indirectly impact reproductive health.^27^ While most research to date centers on gastrointestinal microbiome changes under microgravity and radiation stressors, emerging evidence underscores the importance of site-specific reproductive tract microbiomes—particularly vaginal, endometrial, and uterine—in reproductive outcomes, where dysbiosis has been associated with recurrent implantation failure, infertility, and adverse pregnancy outcomes.^28^^,^^29^ Thus, alterations to these reproductive tract microbial communities should be considered when evaluating how spaceflight-induced microbiome perturbations may indirectly impair reproductive success.

Collectively, reproductive health is equally critical as other well-established priorities in space medicine, such as cardiovascular or musculoskeletal health, because sex-steroid signaling integrates bone, muscle,^30^ cardiovascular,^31^ immune,^32^ and neurobehavioral^33^ performance during flight. Future directions and potential mitigation strategies, such as artificial gravity (AG),^34^^,^^35^^,^^36^^,^^37^^,^^38^ dietary supplements,^39^^,^^40^^,^^41^ and advanced reproductive technologies,^42^ may play an important role in safeguarding reproductive health during long-term space missions and supporting the success of human space exploration (Figure 1). Moreover, microgravity and radiation models uniquely stress telomere,^43^ DNA-repair,^44^^,^^45^ mitochondrial,^46^^,^^47^^,^^48^^,^^49^ and EV pathways,^24^ which may offer translational insights for infertility care, pregnancy loss prevention, oncofertility preservation, and safer cryopreservation practices on Earth. The objectives of this review are to provide an overview of the effects of microgravity on reproductive cells, examine the mechanisms of microgravity-induced cellular stress, and explore the implications for gametogenesis, fertilization, and embryonic development. This review also aims to highlight current experimental studies, identify gaps in knowledge, and propose future research directions to mitigate the risks to reproductive health during long-term space missions.

## Mechanisms of microgravity-induced cellular stress

### Cellular responses to microgravity

Microgravity induces a variety of physiological stress responses in reproductive cells, including oxidative stress, DNA damage, and alterations in cell signaling pathways. The related mechanisms are summarized in Table 1, which highlights the widespread cellular disruptions caused by microgravity and provides a framework for understanding its broader implications for reproductive health. At the molecular level, microgravity shifts redox homeostasis and mitochondrial quality control (↑ROS, mitophagy),^46^ perturbs DNA damage responses (DDRs),^45^ and remodels cytoskeletal^61^ and focal adhesion signaling, which converge on mechanotransducers such as YAP/TAZ.^62^

**Table 1:** Cellular response mechanisms

Mechanism	Normal condition	Reported effect under microgravity	Cell/model examples	Reference
Oxidative stress	Balanced ROS-antioxidant homeostasis	Often ↑ ROS, altered antioxidant enzyme activity, redox imbalance	Germ cells, embryos, fibroblasts	Steller et al.^16^, Dutta et al.^50^, Aitken et al.^51^
DNA repair	Efficient DNA damage sensing and repair	Impaired or delayed repair; accumulation of lesions	Hematopoietic stem/progenitor cells, lymphoid	Beheshti et al.^15^, Low et al.^52^
Cell signaling	Regular MAPK/PI3K-AKT/Ca2+ signaling	Perturbed signaling pathways, altered mechanotransduction, autophagy changes	Germ cells, stem cells, osteoclasts	Jeong et al.^48^, Sambandam et al.^53^
Cytoskeleton	Organized actin-microtubule structures	Disorganization of actin/microtubules, focal adhesion remodeling	Oocytes, embryos, fibroblasts	Crawford-Young^11^, Beck et al.^54^
Mitochondrial function	Normal bioenergetics, stable MMP	Dysfunction, altered OXPHOS, ↑ ROS, changes in ΔΨm/MMP	Oocytes, testicular cells, leukemic cells	Jeong et al.^48^, Santini et al.^55^
EV signaling	Homeostatic secretion and cargo packaging	Altered EV release and cargo (miRNA/protein/lipid); reduced signaling efficacy	Female reproductive tract cells, embryos	Aleksejeva et al.^56^, Machtinger et al.^57^, Simon et al.^58^
Gene expression	Stable transcriptional programs	Altered expression of stress-response and metabolic pathways	Human lymphoblastoid cells, spaceflight twins	Garrett-Bakelman et al.^44^, Chowdhury et al.^59^
Epigenetic status	Maintained DNA methylation and histone marks	Modified DNA methylation, histone PTMs, small RNA changes	Human lymphocytes, lymphoblastoid cells	Chowdhury et al.^59^, Singh et al.^60^

Studies have shown that the unique conditions of space can lead to significant oxidative stress, which adversely affects female reproductive health, driving mitochondrial dysfunction (↓ATP, ↑mitochondrial ROS) and mitophagy via AMPK-mTOR-ULK1/BNIP3 signaling.^47^ Increased oxidative burden coupled with radiation contributes to DNA damage in human cell models such as lymphoblastoid cells and fibroblasts.^15^ Such damage compromises cellular integrity and may, by analogy, impact germ-cell health. Further, impaired DNA repair capacity has been observed in human hematopoietic stem/progenitor cells exposed to simulated microgravity (SMG), leading to long-term genomic instability and raising concerns for reproductive potential.^52^ SMG also impairs DDR kinetics and activates cell-type-specific ATM/ATR-p53 signaling in microglial and glioblastoma cells,^45^ increasing genomic instability in several human lines; hematopoietic progenitors are particularly vulnerable.^52^^,^^63^ Both spaceflight and ground-based simulations consistently indicate that microgravity exacerbates DNA damage and weakens repair capacity, though most direct evidence comes from non-germ systems.^15^^,^^25^^,^^52^

Mitochondrial dysfunction, which is often linked with oxidative stress, has also been reported under space conditions.^16^^,^^25^^,^^55^ It’s a key factor for its contribution to increased production of reactive oxygen species (ROS), exacerbating oxidative damage in oocytes and testicular cells.^55^ Mechanistically, microgravity-associated oxidative stress alters mitochondrial dynamics and bioenergetics across cell types, reinforcing a ROS-mitochondria feedforward loop relevant to oocytes and testicular cells.^47^

Studies in somatic models indicate similar vulnerability. For example, SMG induced mitochondrial dysfunction in human Hodgkin’s lymphoma cells, with ↑ROS and ↓ATP, which triggered autophagy via the AMPK/Akt/mTOR and MAPK pathways.^48^ These same stress nodes (AMPK-mTOR, MAPK) underlie reproductive toxicology and may translate to reduced ovarian reserve or impaired folliculogenesis.^25^

Changes in cytoskeleton and focal adhesions are as follows: microgravity reduces focal adhesions and suppresses FAK/RhoA signaling, altering actin architecture and nuclear positioning; downstream mTORC1/ERK and NF-κB pathways are dampened, promoting apoptosis and defective mechanotransduction.^64^ These adhesion-cytoskeleton changes are a known upstream control for YAP/TAZ, suggesting gravity unloading can shift gene programs via Hippo-independent mechanosignaling.^62^ In oocytes specifically, microgravity disrupts meiotic spindle organization and cortical stability, reducing maturation rates and threatening embryo competence.^65^

EVs are also important mediators of cellular stress responses under microgravity. EVs play a significant role in intercellular communication by carrying bioactive molecules such as proteins, lipids, and RNA that influence recipient cell behavior, including their stress response and overall function.^56^^,^^57^^,^^58^^,^^66^ They are involved in multiple stages of reproduction, from gamete maturation to embryo-maternal communication. By carrying molecules that affect recipient cells, they provide an additional regulatory layer in female reproduction, which relies on finely tuned endocrine processes.^57^ Through intercellular transport, EVs protect molecular cargo from degradation and support essential physiological processes such as folliculogenesis, fertilization, embryo quality, and implantation, all of which are crucial for successful reproduction.^56^^,^^57^^,^^58^ For instance, during the window of implantation, endometrial epithelial-derived EVs promote uterine receptivity and embryo adhesion by delivering specific miRNAs (e.g., miR-30d) to the pre-implantation embryo, which upregulates adhesion/implantation genes and enhances embryo attachment.^67^ Additionally, EVs from the endometrium and embryo also carry proteins, lipids, and small RNAs that modulate extracellular matrix remodeling, integrin/adhesion pathways, and cytokine signaling—key axes of apposition, adhesion, and invasion.^68^ Their properties are influenced by hormonal changes and the health status of reproductive tissues, and they have potential use as biomarkers and therapeutic agents in assisted reproductive technology (ART).^56^ Hormonal milieu (estrogen/progesterone) shapes EV release and cargo across the cycle, aligning EV signaling with the receptive endometrium.^69^ In reproductive fluids (e.g., uterine/follicular fluid), EV cargo—including small RNAs—tracks ovarian physiology^70^^,^^71^ as well as endometrial receptivity^22^^,^^72^ while also predicts the outcomes of ART,^73^ supporting their value as biomarkers and therapeutic candidates.

Under simulated^24^ and real^74^ microgravity,^75^ studies in non-germ cell types (e.g., breast^24^^,^^75^ and thyroid^74^ cancer models) report alterations in EV biogenesis, release, size distribution, surface phenotypes, and miRNA cargo, indicating stress-responsive re-wiring of paracrine networks. While these findings do not derive from germ cells directly, they suggest pathways through which EV remodeling might also perturb uterine receptivity and implantation in reproductive contexts. Accordingly, EV-focused endpoints (release, cargo, and uptake) should be integrated with mitochondrial and DNA-damage/repair readouts and with implantation-relevant signaling/mechanotransduction markers documented in endometrium-embryo models—FAK phosphorylation and the integrin-fibronectin axis, MAPK activation, and SMAD2/3-N-cadherin signaling—to resolve microgravity risk to gamete competence, embryo-maternal crosstalk, and implantation success.^22^^,^^68^ Given their central role in reproductive signaling and cellular stress adaptation, studying EVs under microgravity conditions is essential. Alterations in EV production, cargo, or uptake may disrupt the finely tuned signaling networks that govern reproductive success. Accordingly, integrating EV profiling with mitochondrial/DDR readouts and mechanosensitive markers (FAK-RhoA-YAP/TAZ) may best resolve microgravity risk to germ cells and embryos.

### Effects on cell division and differentiation

The effects of microgravity on cell division and differentiation are highly context-dependent, varying with cell type, experimental timeline, and specific study conditions. While some studies report that microgravity can impair cell division and differentiation, others—particularly in reproductive germ cells—show evidence for the opposite. This complex and sometimes contradictory landscape underscores the need for cautious interpretation and further investigation.

For example, increased DNA damage under microgravity has been associated with disrupted cell cycle regulation, which can lead to genomic instability and affect both mitotic and meiotic divisions.^15^^,^^25^^,^^52^ In reproductive contexts, microgravity’s influence on meiosis and mitosis remains an important concern. Studies have shown that altered cell cycle dynamics under microgravity may impact embryonic development. For instance, exposure of pregnant mice during early gestation resulted in failure to produce viable offspring, suggesting that perturbations in cell division can have downstream reproductive consequences.^76^ Early embryo and oocyte studies directly show spindle defects and reduced maturation under SMG, with suppression of FAK/RhoA focal adhesion signaling under unloading.^64^

Embryonic stem cell studies reveal a dual nature of microgravity’s impact. Embryonic stem cells exposed to spaceflight microgravity showed inhibited differentiation into several tissue lineages, yet exhibited increased cardiomyocyte differentiation upon return to normal gravity.^77^ This bidirectional impact illustrates that microgravity may suppress or facilitate differentiation depending on lineage specificity and post-exposure conditions. Such findings highlight the potential of microgravity as both a challenge and an opportunity in regenerative medicine applications, where controlled differentiation is a key objective.^78^ Comparable lineage biases occur in other systems (e.g., osteogenic pathways are sensitive to integrin/FAK/MAPK suppression), highlighting microgravity’s control over fate via adhesion-dependent signaling.^79^

In germ cell research, SMG has been shown to promote differentiation and meiotic entry in postnatal mouse male germ cells. Specifically, upregulation of markers such as *Kit* (also known as CD117), *Stra8* (stimulated by retinoic acid gene 8), *Scp1* (synaptonemal complex protein 1), and *Spo11* (sporulation protein 11) under SMG suggests that certain reproductive cell types may be primed toward differentiation under these conditions.^80^ This contrasts with observations in other stem cell types where differentiation is suppressed, again reinforcing the cell-specific nature of microgravity’s effects.

SMG has also been associated with impaired differentiation in other types of stem cells. For example, studies using human hematopoietic stem/progenitor cells suggested that SMG impairs DNA damage repair and inhibits their differentiation into dendritic cells, which play a key role in immune function.^52^ In bone cells, increased autophagy has been marked by upregulation of *Atg5* and LC3 (microtubule-associated protein 1 light chain 3), which facilitates osteoclastogenesis.^53^ Although these findings are not directly linked to reproductive health, similar autophagic pathways may play roles in reproductive tissues. For instance, vascular endothelial cells under clinorotation showed autophagy-driven migration changes regulated through HDM2-p53-mTOR signaling, indicating systemic cellular responses to altered gravity.^81^

Interestingly, 4-acetylantroquinonol B (4-AAQB), a compound from *Antrodia cinnamomea*, has been found to inhibit osteoclastogenesis under SMG by suppressing key autophagy proteins (LC3B and p62) and promoting apoptosis.^82^ This suggests that 4-AAQB could offer protective effects in cells where autophagy is similarly activated by altered gravity conditions. Additionally, broader reviews of both SMG and spaceflight studies indicate that microgravity alters differentiation and growth in both normal and cancer stem cells, impacting key pathways like PTEN/FOXO3/AKT and inducing unique behaviors such as spheroid formation and altered gene expression profiles.^83^ These findings carry significant implications not only for reproductive health but also for cancer biology and tissue engineering in microgravity-adapted environments.

While some studies have documented microgravity-induced inhibition of cell differentiation and increased DNA damage, others—especially in reproductive germ cells—report enhanced differentiation under similar conditions. The inconsistency in findings likely reflects differences in cell types studied, exposure durations, gravitational simulation methods, and downstream analysis techniques. Therefore, generalized conclusions should be avoided, and future research would be supposed to delineate the conditions under which microgravity exerts beneficial versus detrimental effects on cellular differentiation and reproductive function.

## Microgravity and gametogenesis

### Spermatogenesis in microgravity

Microgravity significantly impacts spermatogenesis, influencing sperm development, morphology, and motility. Table 2 summarizes the specific effects of microgravity on key reproductive parameters, highlighting reductions in sperm motility, sperm count, and testosterone levels. The conditions of spaceflight microgravity can impair sperm motility and change sperm morphology in rodent sperm/testis models, raising important concerns about fertility in space. Key structural changes in sperm, such as increased DNA fragmentation and compromised acrosome integrity, present serious challenges for successful fertilization under microgravity.^20^ Research shows that microgravity, combined with exposure to ionizing radiation during spaceflight, further worsens sperm function and integrity. Specifically, microgravity reduces sperm motility, overall sperm count, and testosterone levels, while also increasing DNA fragmentation and decreasing testicular weight in rodent models.^84^ Furthermore, oxidative stress is a key factor affecting sperm function and integrity under space conditions, with excessive ROS contributing to DNA damage, decreased motility, and impaired fertilization capacity in human spermatozoa.^50^^,^^51^ Because routine spermiograms can miss chromatin defects, concise molecular endpoints (e.g., sperm nuclear basic protein profiles, protamine-histone ratios, and DNA-binding assays) are required; pollutant-linked alterations in these parameters have been demonstrated in humans^88^ and implicate chromatin-organizing proteins (histones/protamines) in oxidative DNA damage.^4^ Briefly, hyposalinity models also show chromatin vulnerability: in *Mytilus galloprovincialis*, hyposaline exposure upregulates hsp70, down-modulates protamine-like genes, and alters PL-DNA binding in spermatozoa.^89^

**Table 2:** Effects on reproductive parameters

Parameter	Ground Control	Microgravity	Cell/Model Examples	Effect Size/Notes	Reference
Sperm motility	Normal	Significantly decreased	Human/rodent sperm	↓20%–40%	Boada et al.^8^, Mishra and Luderer^10^, Gimunová et al.^12^
Sperm count	Normal	Reduced	Rodent sperm	↓ total count; variable by species and duration	Dutta et al.^50^, Ahrari et al.^84^
Testosterone levels	Balanced	Decreased	Rodent serum/human data	↓ ∼15%–30% reported in spaceflight	Ronca et al.^9^, Gimunová et al.^12^
Oocyte quality	Normal morphology	Compromised structure, mitochondrial changes	Mouse/porcine oocytes	Abnormal MMP↑, OXPHOS↑, meiotic progression altered	Tanghe et al.^85^, Da Broi et al.^86^
Follicle development	Normal growth	Impaired folliculogenesis	Mouse ovarian follicles (SMG)	↓ secondary follicles; disrupted gap junctions	Cheng et al.^87^
Hormone regulation	Balanced	Dysregulated	Human/rodent reproductive axis	Altered LH/FSH/E2 regulation	Ronca et al.^9^, Gimunová et al.^12^
DNA integrity	Stable	Increased DNA fragmentation	Human/rodent sperm cells	↑ DNA strand breaks, ↑ γ-H2AX foci	Li et al.^14^, Beheshti et al.^15^, Steller et al.^16^
Fertilization potential	Normal	Reduced	Mouse/rat embryos	↓fertilization and cleavage rates	Machtinger et al.^20^^,^^57^, Simon et al.^58^

EVs play a significant role in supporting sperm maturation and function. Various types of EVs, including those found in seminal, follicular, and uterine luminal fluids, contribute to the regulation of key reproductive processes.^20^ In seminal plasma EVs, vesicles facilitate post-testicular sperm maturation by aiding in sperm motility acquisition and reducing oxidative stress, both of which play essential roles in maintaining sperm functionality.^90^^,^^91^ As sperm transit through the epididymis, they undergo maturation, acquiring the capacity for fertilization. This process is regulated by EVs containing essential proteins and miRNAs that help in the functional development of sperm.^92^^,^^93^ The importance of EVs is further emphasized by the fact that sperm cells, due to their compact structure and lack of organelles necessary for *de novo* protein synthesis, rely heavily on these extrinsic signals for functional competence.^93^^,^^94^ The presence of EVs in seminal plasma plays a regulatory role in maintaining the morphofunctional characteristics of spermatozoa, potentially mitigating the negative effects of oxidative stress by regulating sperm maturation and providing antioxidant support.^95^^,^^96^ More recently, seminal EVs from humans were shown to improve sperm function via CatSper-mediated Ca^2+^ signaling.^97^

Because simulated and real microgravity remodel EV biology (Section cellular responses to microgravity)—altering EV release, surface phenotypes, and miRNA cargo in non-germ models—it is plausible that epididymal/seminal EVs will likewise be perturbed, reducing EV-mediated signaling efficiency. Direct microgravity data on male reproductive-tract EVs are currently lacking; however, given their established roles in capacitation and CatSper-mediated Ca^2+^ signaling, shifts in epididymal/seminal EV abundance or cargo could plausibly impair sperm function in space. Near-term countermeasures include antioxidant strategies to limit ROS-mediated damage^50^^,^^51^ and cautious exploration of EV-based interventions (e.g., supplementation with EVs that enhance CatSper-mediated Ca^2+^ signaling).^84^^,^^97^

To increase interpretability and avoid overgeneralization, future work should explicitly distinguish germ-cell from non-germ EV responses. Specifically, targeted studies are needed to determine whether epididymal and seminal plasma EVs alter their release, cargo, or CatSper-activating potential under microgravity, and whether EVs from cumulus-oocyte complexes or uterine fluid experience comparable changes that could impact capacitation, fertilization, or embryo-maternal communication. Finally, situating spaceflight within the broader continuum of reproductive stressors, evidence from polluted terrestrial settings shows that pollution-related trace elements and other exposures are associated with altered semen composition and quality, supporting human semen as an early, sensitive biomarker of environmental impact on male fertility; adopting analogous molecular endpoints (e.g., SNBP profiling, protamine-histone ratios, and DNA-binding assays) can strengthen assessments of microgravity-exposed sperm.

### Oogenesis in microgravity

Microgravity also impacts oogenesis, affecting oocyte maturation, structure, and fertilization potential. Recent work suggests that SMG induces a distinctive mitochondrial phenotype in mouse oocytes— enhanced oxidative phosphorylation (OXPHOS), abnormal mitochondrial membrane-potential (MMP) hyperpolarization, and mitochondrial mislocalization—accompanied by accelerated meiotic progression, delayed MTOC coalescence, spindle defects, increased aneuploidy, and reduced developmental potential; prolonging M-phase (APC inhibition) partially rescues maturation.^49^ Because spindle positioning and oocyte polarity are essential for asymmetric division and faithful chromosome segregation,^98^ their disruption under microgravity directly compromises cytoplasmic partitioning and genomic integrity, thereby diminishing fertilization success and subsequent embryonic competence. Together with prior flight/analog observations of ovarian disruption,^10^ these findings support mitochondria-centered mechanisms linking altered mechanics to oocyte-quality loss under microgravity.

The cumulus oophorus, which surrounds the oocyte, plays a crucial role in oocyte maturation, ovulation, and fertilization through mechanisms such as metabolic cooperation, sequestration of sperm, and induction of the acrosome reaction, providing essential nutrients and signaling molecules.^85^ In microgravity, changes in cumulus cell function may impair the delivery of essential factors to the oocyte, potentially affecting its quality and maturation. Cumulus cells, which are closely associated with the oocyte, are important for providing metabolic support and are involved in regulating meiotic resumption and cytoplasmic maturation,^99^ compromised cumulus function under altered gravity could lead to abnormalities in oocyte maturation, reducing the chances of successful ovulation.^85^^,^^86^

In addition, EVs present in follicular fluid of bovine, porcine, human, and murine species play a significant role in regulating follicular growth, oocyte maturation, and steroidogenesis, all of which are essential for successful fertilization.^20^^,^^71^ These EVs facilitate communication between the oocyte and surrounding granulosa cells, supporting follicular growth and oocyte competence. For instance, EVs carry bioactive molecules such as miRNAs that regulate key pathways involved in oocyte maturation and follicular development.^20^ Although direct data under microgravity are lacking, by analogy to SMG-induced EV remodeling in non-germ systems (Section cellular responses to microgravity), disruption of EV signaling in space could impair this oocyte-granulosa communication, reducing oocyte quality and decreasing fertilization potential.^71^^,^^100^

Microgravity’s influence on ovarian function also extends to hormonal regulation, potentially disrupting the fine-tuned endocrine balance required for reproductive success. Hormonal dysregulation can lead to altered steroidogenesis, which may impair oocyte quality and hinder successful ovulation.^10^ Evidence from SMG in mouse ovarian follicles has shown decreased oocytes quality due to disrupted communication structures between granulosa cells and the oocyte, including transzonal projections and microvilli, which are essential for nutrient and signal exchange.^87^ Mechanistically, the combined impact of altered cumulus function, disrupted EV signaling, hormonal imbalance, and SMG-driven mitochondrial hyperactivity/MMP hyperpolarization in oocytes (with downstream spindle and MTOC defects) under microgravity conditions outlines a coherent pathway for reduced oocyte competence in space. Importantly, M-phase timing control has emerged as a potential mitigation lever, highlighting both the risks and opportunities for safeguarding oogenesis and fertilization in space environments.

## Molecular and epigenetic changes in reproductive cells

### Gene expression alterations

Exposure to microgravity is associated with notable changes in gene expression, especially in genes associated with cell cycle regulation, DNA repair, and stress response pathways. Spaceflight and ground-based simulations have been shown to perturb cellular structures and transcriptional programs in ways that could impair embryonic development, potentially resulting in issues such as neural tube defects, morphological abnormalities, and even embryo loss.^11^ Cytoskeletal components, such as microtubules and actin filaments, are especially sensitive. For instance, mouse fetal fibroblasts chronically exposed to SMG showed remodeling of the cytoskeleton and adhesion machinery, accompanied by altered expression of actin-related genes, including *Actg2* (actin gamma 2), *Acta1* (actin alpha 1), and *Fhl1* (four and a half LIM domains 1), in part via the Rho signaling pathway.^54^ These findings are consistent with broader observations of rapid actin/cytoskeletal changes under microgravity in multiple non-germ cell types, including muscle and endothelial cells.

Cytoskeletal integrity is crucial for cellular processes such as migration, division, polarity, and differentiation; its disruption can misposition embryonic cells, distort tissue patterning, and compromise structural stability, ultimately contributing to developmental challenges. Additionally, studies have indicated that changes in the cytoskeleton can also interfere with cell polarity and intracellular transport, which are essential for normal embryogenesis.^101^^,^^102^

Microgravity perturbs cytoskeletal architecture through well-defined mechanotransduction nodes, notably the integrin-FAK–RhoA/ROCK axis and its downstream links to focal adhesions and microtubule/actin dynamics, leading to reduced focal adhesion signaling, cytoskeletal disorganization, and altered nuclear positioning^61^^,^^103^; these effects have been documented across multiple cell types and are mechanistically consistent with force transmission to chromatin via the LINC complex and the nuclear lamina.^104^^,^^105^ In germ cells, these pathways have direct consequences: oocyte maturation and early embryonic cleavages rely on precise actin-cortex and microtubule spindle positioning for asymmetric division, pronuclear migration, and faithful chromosome segregation.^106^ Simulated microgravity disrupts meiotic spindle organization and induces cortical blebbing in mouse oocytes, while recent work highlights that spindle stability near the cortex depends on active cortex-cytoplasm coupling that is sensitive to changes in intracellular flows—conditions likely altered in microgravity.^65^ In addition, microgravity-associated mitochondrial and oxidative stress responses in oocytes can arrest maturation (Section oogenesis in microgravity), providing a bioenergetic route by which cytoskeletal control of meiosis fails.^49^

The effects of microgravity extend beyond cytoskeletal alterations. Evidence from both spaceflight and simulated experiments shows that microgravity, especially when combined with space radiation, induces oxidative stress, which can disrupt DNA repair pathways and increase cellular susceptibility to genetic damage, ultimately compromising cellular stability and function.^15^ Chronic exposure to SMG in mouse fibroblasts has been shown to primarily impact oxidative stress responses, marked by increased expression of genes such as *Gsta1* (glutathione S-transferase alpha 1), *Gsta2* (glutathione S-transferase alpha 2), and *Hmox1* (heme oxygenase 1), under the transcription factor Nrf2 control.^54^

Importantly, in-flight and post-flight responses are not identical. Multi-omic studies, including the NASA (National Aeronautics and Space Administration) Twins Study,^107^ reveal phase-specific biological trajectories—for instance, telomeres lengthen during spaceflight but shorten rapidly after return, with some deviations persisting for months.^43^ Oxidative stress, a key contributor to cellular aging and damage, is consistently upregulated in space, indicating an intensified cellular defense mechanism that may paradoxically promote premature cellular senescence and impaired repair functions. Garrett-Bakelman et al. (2019) further reported lasting changes in immune and DNA-repair gene regulation in astronauts, with some persisting months after return to Earth.^44^ Crucially, most astronaut telomere measurements are derived from peripheral blood leukocytes and do not assess telomerase activity in germ cells; germ cells and early embryos exhibit distinct telomere biology with higher or developmentally reactivated telomerase compared with most somatic tissues.^108^^,^^109^ Therefore, somatic telomere dynamics observed in-flight should not be overgeneralized to germline without direct data.

Complementing these insights, recent proteomic analyses of astronaut plasma show that many protein changes first appear in-flight, particularly affecting pathways tied to immunity, cytoskeletal organization, and coagulation. However, distinct re-adjustments occur post-flight, including increases in proteins such as BASP1 and IGFBP4 at 90 days after return, consistent with signatures of re-adaptation to Earth’s gravity.^110^ These observations underscore that many post-flight shifts are not adaptations to microgravity but re-adaptation responses to terrestrial conditions. Therefore, in-flight cellular responses must be carefully distinguished from post-flight re-adaptation, a distinction important for accurately interpreting risks to reproductive health in space. In the specific context of reproduction, early embryos can lengthen telomeres through telomerase and recombination-based mechanisms during zygotic genome activation and toward the blastocyst stage,^111^^,^^112^ further underscoring the need for germ cell—and embryo—specific measurements under space conditions.

### Epigenetic modifications and long-term genetic implications

Epigenetic changes, including DNA methylation, histone modifications, and non-coding RNA expression, have been observed in response to spaceflight. These modifications regulate gene activity without altering DNA sequence and can influence cellular stress responses. In some contexts, these epigenetic states can persist beyond the exposed individual and influence offspring phenotypes.^113^

SMG studies in human non-germ cells have shown widespread DNA methylation and hydroxymethylation changes, alongside transcriptional reprogramming. For example, human lymphoblastoid cells showed altered 5mC/5hmC patterns in pathways related to oxidative stress, metabolism, and transcriptional regulation,^59^ accompanied by coordinated gene-expression shifts.^114^ Similarly, human lymphocytes exhibited upregulation of DNA methyltransferase genes, such as *DNMT1*, *DNMT3A*, and *DNMT3B*, which has been linked to DNA hypomethylation and genomic instability.^60^ Histone marks can also be gravity-sensitive: in murine thymocytes, gravitational stress remodeled H3K27me3/EZH2 activity, illustrating that chromatin writers/erasers respond to altered loading conditions (albeit in somatic cells).^114^

Human spaceflight evidence reinforces these observations. The NASA Twins Study indicated significant changes in DNA methylation, gene expression, and telomere dynamics during long-duration missions.^44^ Telomere length initially increased during spaceflight but rapidly shortened post-flight, likely driven by oxidative stress and DNA damage responses.^115^ At the embryo level, true spaceflight exposure produced blastocysts with global hypomethylation and unique DMRs, together with increased DNA damage, directly tying epigenetic dysregulation to early developmental risk.^116^ Importantly, telomerase regulation differs fundamentally between somatic and germ cells (and across preimplantation stages), with measurable telomerase activity in spermatogonia and reactivation in early embryos^108^^,^^117^; this biological difference cautions against equating somatic telomere changes with germline consequences in the absence of germ cell-specific assays (e.g., TRAP)^108^ performed under microgravity or spaceflight.

Spaceflight elicits conserved miRNA responses across rodents and humans (a “spaceflight miRNA signature”), implicating post-transcriptional regulation of DNA-repair, immune, and mitochondrial pathways; these ncRNA shifts may intersect with reproductive pathways and could be tracked in germline-adjacent biofluids.^118^ Given their stability and regulatory breadth, miRNAs are attractive epigenetic biomarkers/countermeasure targets during missions.

Genetic background further modulates these risks. Variants in DNA repair genes may influence individual susceptibility to radiation and microgravity, highlighting the possibility of heritable genetic damage.^15^ Moreover, epigenetic alterations may themselves be heritable,^25^ raising the possibility of intergenerational impacts on reproductive health and developmental outcomes.

In mice, paternal spaceflight has been linked to intergenerational epigenetic effects: short-term ISS exposure altered ATF7-dependent chromatin states in testes and changed the small-RNA (miRNA) payload of sperm; F1 offspring of space-traveling males showed altered hepatic transcriptomes, consistent with sperm-borne epigenetic information transfer.^119^ These findings complement embryo-level spaceflight data showing global hypomethylation and distinct DMRs in space-grown mouse blastocysts.^116^ Evidence for persistence beyond F1 (true transgenerational inheritance) under microgravity is currently limited; simulated-microgravity models (e.g., hindlimb unloading) demonstrate systemic molecular shifts^120^^,^^121^ but lack definitive multi-generation germline methylome/small-RNA maps. Accordingly, while mouse data support intergenerational epigenetic transmission after spaceflight, multi-generational persistence and human relevance remain to be established.

Together, these findings underscore both the genetic and epigenetic consequences of spaceflight and SMG. The persistence of some alterations, such as DNA damage, telomere length changes, and epigenetic modifications, suggests that long-term space missions may pose heritable risks, though much of the current evidence derives from non-germ cells. To close the gap, we recommend: (i) germline-focused methylome/hydroxymethylome mapping (bisulfite/oxBS-seq), histone-mark ChIP-seq (e.g., H3K27me3/H3K9me3/H3K27ac), and ncRNA (miRNA/piRNA) profiling in validated microgravity platforms; (ii) embryo-stage-specific analyses (zygote→blastocyst) to couple DMR/mark changes with lineage allocation and DNA-damage repair; and (iii) integration with spaceflight miRNA signatures for biomarker-guided countermeasures.

## Effects on fertilization and early embryonic development

### Challenges in fertilization

Microgravity conditions have been shown to reduce fertilization rates, potentially due to altered sperm-egg interactions and impaired zygote formation. Lei et al. (2019) investigated the effects of microgravity on mammalian reproduction and found that the cleavage of mouse pre-implantation embryos and blastocyst lineage formation were significantly disrupted under both spaceflight and SMG conditions.^7^ In parallel, spaceflight experiments suggested that mouse 2-cell embryos can reach the blastocyst stage in orbit, but blastocyst yield and quality are reduced and accompanied by DNA damage and global hypomethylation, underscoring additional gravity-linked vulnerabilities at the embryo stage.^116^ These findings highlight the complexity of fertilization and the key role of gravitational forces in facilitating successful sperm-egg interactions and subsequent zygote development.

The cumulus oophorus supports fertilization by promoting metabolic cooperation, sequestering sperm, and inducing the acrosome reaction during oocyte maturation, ovulation, and fertilization.^85^ Under microgravity, the physical and biochemical interactions between the cumulus cells and sperm may be altered, potentially impairing the acrosome reaction and reducing the fertilization efficiency. Their role has been elucidated by Van Soom et al. (2002), who found that removal of the cumulus often leads to decreased fertilization rates.^113^ The cumulus may guide sperm toward the oocyte, create a favorable environment for sperm capacitation, and prevent unfavorable changes in the oocyte. Consistent with a gravity-sensitive cumulus-oocyte complex (COC), SMG disrupts granulosa-cell transzonal projections and oocyte microvilli (Oo-Mvi) that mediate germline-somatic communication, lowering oocyte quality; supplementation with the oocyte-secreted factor GDF9 partially rescues these defects.^87^ Complementing this, human metaphase-II oocytes exposed to SMG show ultrastructural perturbations in mitochondria, endoplasmic reticulum, and cortical granules—changes predicted to diminish subsequent fertilization competence.^122^

Beyond the COC, the oviductal epithelium—particularly its motile cilia—coordinates oocyte pick-up, sperm reservoir dynamics, and gamete/embryo transport. Genetic and imaging studies in mice show that motile cilia in the infundibulum are essential for oocyte pick-up and that ciliary function contributes region-specifically to transport and fertility.^123^^,^^124^ Although oviductal cilia under microgravity have not been tested directly, ciliated structures in other tissues are gravity-responsive (e.g., primary cilia shorten and alter trafficking under SMG), suggesting a plausible vulnerability of cilia-mediated transport in space.^125^

EVs have also been identified as important contributors to successful fertilization. EVs present in seminal, follicular, and uterine luminal fluids carry signaling molecules, such as miRNAs, that regulate sperm maturation, enhance oocyte competence, and prevent polyspermy.^20^ These EVs help create an optimal environment for fertilization, and their compromised function during spaceflight could negatively impact fertility. Harris et al. (2020) indicated that oviduct-derived EVs (oEVs) improve fertilization and early embryo development by modulating gamete interactions and preventing polyspermy.^126^ These oEVs are incorporated into gametes and embryos, supporting fertilization, enhancing oocyte maturation, and improving embryo quality, underscoring their importance for reproductive success under challenging conditions like microgravity. Additionally, Bridi et al. (2020) highlighted that small EVs from the oviduct, endometrium, and embryos are essential for embryo-maternal communication necessary for pregnancy establishment.^127^ Importantly, microgravity perturbs EV biology in other cell types—both simulated and real microgravity alter EV release and cargo—raising concern that reproductive-tract EV signaling could likewise be dysregulated during spaceflight.^24^^,^^128^ Taken together, these cellular (cumulus/granulosa and oviductal) and EV-mediated support systems provide concrete mechanisms by which microgravity could impair sperm-oocyte communication and fertilization, even when individual gametes remain viable.

### Embryo development in microgravity

Microgravity has also been found to negatively impact early embryonic development, leading to abnormalities in embryo structure, cell polarization, and implantation potential. Lei et al. (2019) showed that microgravity, considering both real and simulated space conditions, affects the cleavage of mouse pre-implantation embryos and interferes with blastocyst lineage formation, which is crucial for successful implantation.^7^ Furthermore, Lei et al. (2020) suggested that mouse preimplantation embryos in space exhibited compromised blastocyst quality, DNA damage, and global hypomethylation, implicating that both microgravity and space radiation can contribute to developmental defects.^116^ Wakayama et al. (2009) also reported impaired mouse embryo development under microgravity from a three-dimensional (3D) clinostat, with reduced trophectoderm cell numbers, although some embryos developed to term.^129^ Specifically, compared with 1 G controls, microgravity-cultured blastocysts had significantly fewer trophectoderm (TE) cells (mean 5.9 vs. 17.0 per embryo) while inner cell mass numbers were similar (mean 8.0 vs. 8.7), and blastocyst formation after 96 h was lower (30% vs. 57%). *In vivo* viability was also reduced, with live-birth rates of 16% vs. 37% for blastocyst transfers and 35% vs. 63% for 2-cell transfers; importantly, cellular polarity—reflected by preserved inner cell mass/trophectoderm (ICM/TE) localization—was maintained despite fewer TE cells (consistent with reduced TE differentiation) under clinostat conditions.^129^ Such disruptions in the early stages of development could compromise embryo viability and reduce developmental success.

Mitochondrial dysfunction and oxidative stress represent additional risks. Cryopreservation-induced mitochondrial dysfunction in reproductive cells (oocytes/embryos) has been shown to disrupt fertilization and impair embryo development.^130^ Under microgravity, similar mitochondrial challenges are anticipated due to increased oxidative stress and impaired mitochondrial function observed in space. Harvey (2019) noted that deficits in mitochondrial function can result in reduced oocyte and embryo quality, along with post-implantation failure.^131^ May-Panloup et al. (2021) emphasized the central role of mitochondria in early embryonic development, as their dysfunction disrupts energy production, cell signaling, and embryo viability.^132^ As mitochondria supply the energy required for cleavage, cell polarity, and differentiation, their dysfunction under microgravity could have notable impacts on implantation success and embryo viability.

## Current experimental studies and models

Understanding the effects of microgravity on reproductive health requires a multi-faceted approach, employing diverse research methodologies. Table 3 categorizes these methodologies, including *in vitro* simulations, animal studies, and human research, detailing key findings from each. Together, these approaches offer structured insights into how biological systems adapt to or are disrupted by altered gravity. The following subsections delve into these methodologies, examining their key findings and the role they play in advancing our knowledge of reproductive health challenges in space.

**Table 3:** Research methodology classification

Study Type	Model System	Key Findings	Reference
*In vitro* simulation	Clinostat/RPM	↑ Apoptosis, DNA damage	Prasad et al.^133^, Hada et al.^134^, Dang et al.^135^
	RCCS	Mitochondrial dysfunction	Li et al.^14^, Singh et al.^136^
	3D clinostat	Chromosome aberrations	Beck et al.^137^, Hada et al.^134^
*In vivo* studies	Rodent models	Testicular injury, ↑ apoptosis in germ cells	Jennings and Santy^6^, Lei et al.^7^, Li et al.^76^, Liu et al.^138^
	Mouse embryos	Developmental abnormalities	Lei et al.^116^, Wakayama et al.^129^
	Sperm analysis	Reduced fertility	Dutta et al.^50^, Ahrari et al.^84^

In addition, different devices and approaches have been developed to simulate or directly study microgravity. Table 4 provides a comparative overview of these approaches, highlighting their principles, advantages, limitations, and representative applications. This summary enables a clearer understanding of how various experimental systems contribute complementary perspectives to space biology research.

**Table 4:** Comparative overview of microgravity approaches

Approach/device	Principle (brief)	Pros	Cons	Brief example	Reference
2D clinostat	Single-axis rotation to time-average g	Simple, accessible	Residual g; rotation-induced shear	Endothelial cells under clinorotation	Li et al.^81^
RPM/3D clinostat	Random/dual-axis rotation averaging g-vector	Longer exposures feasible	Settings-dependent outcomes	SMG reports of apoptosis/DNA damage	Prasad et al.^133^
RCCS (rotating wall)	Fluid co-rotation → low-shear suspension	Supports 3D spheroids	Nutrient gradients	Stem/cancer stem cell literature	Grimm et al.^83^
SMG + radiation	SMG device + ionizing radiation	Models multi-stressor space milieu	Hard to deconvolve factors	Spermatogenic cell apoptosis/DNA damage	Li et al.^14^
Spaceflight (ISS)	True microgravity in orbit	Highest ecological validity	Small N; logistics constraints	Mouse preimplantation embryos in flight	Lei et al.^116^

### *In vitro* studies and simulated microgravity

Various models of SMG have been utilized in laboratory studies to examine its effects on reproductive cells and related biological systems. Among the most widely used are clinostats, which slowly rotate samples to average the gravity vector; random positioning machines (RPMs), which continuously reorient samples in three dimensions to nullify the net gravitational pull; and rotating wall vessels (RWVs) and rotary cell culture systems (RCCSs), which are low-shear bioreactors that keep cells in gentle suspension. Each platform operates on distinct principles and comes with specific advantages and limitations. Clinostats (2D or 3D) and RPMs (a type of 3D clinostat with randomized rotation) simulate microgravity by constantly changing the direction of gravity’s pull.^133^ This approach effectively nullifies gravity in a small central volume of the device, making it suitable for small samples like cell cultures. Clinostat-based systems are relatively simple and cost-effective, allowing long-duration experiments; however, they do not truly eliminate gravity—the force is averaged rather than removed—so cells may still experience residual gravitational effects and mechanical stress from rotation.^139^ RPMs improve upon this by using multi-axial rotation to achieve high-quality microgravity (down to ∼10^−4^ g) in the lab. Even so, RPM experiments must be carefully controlled, as rotation can induce fluid motion and shear stress in the culture, potentially affecting nutrient delivery and cellular responses. In contrast, RWV/RCCS bioreactors are horizontally rotated culture vessels that balance the sedimentation speed of cells, keeping them gently suspended in media.^133^ This creates a low-turbulence, low-shear environment that encourages three-dimensional cell aggregation (spheroid formation) and provides a microgravity analog with minimal mechanical disturbance. A key advantage of the RCCS is its ability to culture suspension or anchorage-dependent cells on microcarrier beads in a near free-fall state, which often leads to more physiologically relevant 3D cell models. On the other hand, RWVs are limited by diffusion gradients in larger spheroids and require careful adjustment of rotation speed to prevent cells from either settling or experiencing excess shear.

Experimental outcomes under simulated microgravity (SMG) vary depending on the device and culture context, as each platform imposes distinct mechanical forces, fluid shear, and mass transport conditions. In some studies—particularly when SMG is combined with radiation or assessed in vivo—SMG has been shown to exacerbate cellular stress responses, such as increasing DNA damage and apoptosis.^138^ However, findings diverge across platforms. In RCCS, SMG consistently amplifies spermatogenic apoptosis, sperm DNA damage, and chromatin structure alterations,^14^ whereas it contributes to mitochondria-mediated apoptosis and changes in DNA damage response protein expression among HL-60 promyelocytic leukemic cells.^136^ In rotating wall vessel bioreactor, it intensified radiation-induced apoptosis via ROS-sensitive pathways in B-lymphoblasts.^135^ Similarly, in 3D clinostat systems, SMG during radiation exposure yields more chromosome aberrations in human fibroblasts, suggesting combined genotoxic stress.^134^ By contrast, RPM-based studies show less uniform trends. Some report mitigating effects, such as reduced apoptosis in irradiated fetal fibroblasts, as indicated by caspase activity assessments.^137^ Yet in TCam-2 male germ cells, RPM exposure sustains ROS/Ca^2+^ dysregulation, mitochondrial dysfunction, and oxidative stress responses—partially rescued by antioxidants.^140^

Together, these *in vitro* studies highlight the essential role of various microgravity simulation models in understanding cellular responses. While many findings are derived from non-germ cells, they provide mechanistic insights that may inform hypotheses about germ cell biology under space conditions. Such models remain indispensable for dissecting pathways of DNA damage, apoptosis, and oxidative stress relevant to reproductive health in altered gravity.

### *In vivo* animal models

Animal models, particularly rodents, are essential for studying how altered gravity in space affects reproductive cell function and viability. These *in vivo* studies provide insights into the impact of microgravity on reproductive systems and fetal development.

B. Mishra et al. explored the effects of microgravity on reproductive health using rodent models, exposing them to both spaceflight microgravity and Earth-based simulations.^10^ Their observations revealed disruptions in estrous cycles and follicle development in female rodents, as well as alterations in spermatogenesis and testosterone synthesis, suggesting that microgravity could impair multiple aspects of reproductive physiology. P. Santy and colleagues further examined reproductive challenges in space by reviewing various animal studies.^141^ Their findings emphasized that microgravity alone, even without the added effects of radiation, significantly influences reproductive health and could compromise reproductive viability and function.

Sex-specific sensitivity. Female rodents flown on the ISS displayed estrous-cycle perturbations and ovarian transcriptomic changes after 37 days of spaceflight, indicating cycle dysregulation and altered ovarian signaling^142^ and, in ground analogs, prolonged estrous cycles with reduced estradiol have been reported under hindlimb unloading (HU).^143^ In males, HU models show suppression of the hypothalamic-pituitary–gonadal axis via downregulation of hypothalamic Kiss1 (a key upstream regulator of gonadotropin-releasing hormone [GnRH]), with concomitant impairments in spermatogenesis and testicular function^144^; time-dependent disruption of testicular histology under HU has also been documented.^145^ These data together suggest that female cycling/ovarian endpoints and male hypothalamic-testicular endpoints may exhibit distinct vulnerability profiles to altered gravity.

These *in vivo* methodologies underscore the complexity of assessing microgravity’s impact on reproduction and highlight sex-specific considerations. While rodent models provide crucial evidence, interspecies variability limits direct extrapolation to humans; future work should (i) pre-specify male versus female endpoints (e.g., Kiss1-GnRH-luteinizing hormone [LH]/testosterone and spermatogenesis in males; estrous cyclicity, folliculogenesis, and ovarian gene networks in females), and (ii) integrate rodent findings with human-relevant biomarkers to refine risk models and countermeasures for long-duration missions.

### Human studies and prospective research

There are significant studies shedding light on the effects of altered gravity in space. The NASA Twins Study compared a twin who spent 340 days on the ISS with his Earth-bound brother, revealing gene expression changes related to immune function and oxidative stress, along with telomere elongation during the mission and rapid shortening post-mission, suggesting accelerated aging.^15^ Importantly, these cohorts assessed telomere length primarily in peripheral blood (somatic) cells and did not measure telomerase activity in germ cells; because telomerase is regulated differently in the germline and is developmentally reactivated in early embryos,^108^^,^^112^ extrapolating somatic telomere dynamics to reproductive risk should be done with caution. Complementing these observations, a review of space-induced genomic changes notes that microgravity perturbs oxidative stress response, cell cycle regulation, and immune function, and is linked to epigenetic modifications such as DNA methylation and histone changes.^146^

Beyond these systemic findings, operational reproductive management for women currently relies on contraception and menstrual suppression strategies during training and flight. Historically, many female astronauts have used continuous combined oral contraceptive pills (COCPs) to induce amenorrhea on long-duration missions; NASA materials also note that astronauts have autonomy in method choice, with some selecting levonorgestrel-releasing intrauterine devices (LNG-IUDs) and many using continuous OCPs for both suppression and contraception.^147^ Long-acting reversible contraceptives (LARCs)—including LNG-IUDs and the etonogestrel implant—are emphasized in aerospace and clinical guidance for their high efficacy, adherence independence, and low mass/waste burden, whereas depot medroxyprogesterone acetate and GnRH agonists are additional options used in specific scenarios^147^^,^^148^; selection must weigh mission logistics (resupply, trash, volume) and health trade-offs (e.g., bone mineral density considerations and venous-thromboembolism risk with estrogen-containing OCPs).^149^ For non-contraceptive management, reusable menstrual cups are now being tested under spaceflight conditions as a potential hygiene solution,^150^ though they do not provide contraception.

Importantly, no direct human germ-cell measurements in space have been reported; conclusions are indirect. Research on human reproductive health in space is largely prospective, constrained by the ethical and logistical challenges of conducting such studies in orbit. While current practice includes menstrual suppression and contraceptive use as described above, systematic, astronaut-focused safety and performance data for these methods in microgravity remain limited, highlighting a clear need for prospective registries and standardized reporting.^151^ Despite these challenges, understanding the potential risks to reproductive health is increasingly important, especially with growing interest in long-term space exploration and habitation. The complexity lies not only in the biological impacts, such as increased DNA damage and altered reproductive cell function, but also in the difficulty of obtaining reliable experimental data. While current studies using advanced *in vitro* models and animal studies provide valuable insights, they cannot fully replicate human reproductive systems under space conditions. Ongoing efforts aim to use these models to better predict and mitigate potential risks for astronauts, contributing to the development of safety measures and guidelines for protecting human reproductive health during extended space missions.^15^

## Implications for space biology and human health

### Long-term space missions

Maintaining reproductive health during extended space missions poses significant challenges, as the effects of microgravity on the human reproductive function are still not fully understood. Beyond general endocrine disruption, spaceflight shows sex-specific hormonal patterns that matter for reproductive risk assessment. Factors such as altered atmospheric pressure and the absence of gravitational forces impact reproductive function in space, potentially threatening species survival and crew health. Accordingly, reproductive health should be prioritized alongside cardiovascular health for spacefaring crews, given their shared, hormone-linked effects on mission performance and medical risk.

Microgravity can alter hormone levels and impair sperm quality, affecting both male and female astronauts’ reproductive health.^12^ For men, pooled astronaut data indicate that total, free, and bioavailable testosterone are not significantly different during long-duration ISS flights, but all decrease on landing day (*p* < 0.01), consistent with re-entry/re-adaptation effects rather than persistent in-flight hypogonadism; similar short-duration shuttle patterns and bed-rest analogs support this distinction.^152^ Countermeasure research in head-down bed rest further tests intermittent, low-dose testosterone as an adjunct to exercise, emphasizing careful risk-benefit evaluation rather than routine use.^153^

For women, flight and analog studies point to hypo-estrogenic signatures and disrupted ovarian signaling. Space-flown adult mice showed estrous-cycle arrest, loss of corpora lutea, and reduced uterine estrogen-receptor (ESR1) transcripts; ISS transcriptomics and follow-ups also report altered estrogen-pathway gene expression.^142^ SMG (hindlimb unloading) prolongs diestrus and lowers plasma estradiol in rats, consistent with hypoestrogenism.^154^ Direct progesterone measures in human flight are lacking, but the loss of corpora lutea in flown mice implies reduced luteal progesterone and potential implantation risk—an inference that warrants targeted human studies.^142^ In addition, spaceflight has been shown to alter estrogen receptor expression and signaling in several tissues, including liver, muscle, kidney, skin, and retina, in female mice,^155^ highlighting systemic modulation of estrogen pathways that may also have implications for reproductive health.

Cohabitation of male and female astronauts during long-term missions also raises questions about conception and reproduction in microgravity, including the feasibility of both natural and assisted reproduction, but human germ-cell and sex-steroid datasets remain sparse and should not be over-generalized from somatic endpoints. As mission durations increase and crews age, understanding the long-term effects of microgravity on reproductive health becomes essential to support healthy aging post-mission.^5^ Near-term priorities include: (i) standardized, in-flight profiling of sex steroids (estrogen, progesterone, testosterone) with post-flight follow-up; (ii) ovarian/testicular function readouts (e.g., cycle tracking, LH/follicle-stimulating hormone [FSH], anti-Müllerian hormone) alongside validated analogs; and (iii) evaluation of countermeasures (exercise, pharmacologic support, and AG) with sex-specific safety endpoints.^156^

Taken together, current evidence supports a concise, sex-specific view: men often show landing-day testosterone dips without consistent in-flight suppression, whereas females exhibit microgravity-associated hypoestrogenic features in animal/analog models with altered estrogen-pathway signaling and likely secondary progesterone consequences. Hence, focused human studies are needed to translate these findings to operational guidance for exploration-class missions.

### Future directions and mitigation strategies

To ensure mission reliability and crew safety, effective countermeasures must be developed and applied. Reproductive health intersects with operational safety because sex-steroid hormones (e.g., estrogen, progesterone, testosterone) influence bone, muscle, cardiovascular, immune, and neurobehavioral systems^157^; destabilizing these axes can degrade physical readiness, increase fracture/cardiovascular risk, and create medical contingencies (e.g., unplanned pregnancy management) during exploration-class missions. Accordingly, key strategies include AG, exercise regimens, nutrition-based countermeasures, and advanced shielding.

Diet is a core countermeasure domain for spaceflight and is already managed as part of integrated risk reduction (e.g., tailored macro/micronutrient targets, vitamin D provisioning, and iron monitoring).^40^ Spaceflight elevates oxidative and inflammatory stress; targeted nutrients that support redox balance and mitochondria are therefore plausible adjuncts to AG/exercise.^39^ For male fertility specifically, clinical reviews report benefits of select antioxidants (e.g., vitamins C/E, CoQ10, carnitine, zinc, selenium, folate) on semen parameters when used judiciously^158^; nevertheless, protocols should avoid over-supplementation that can induce reductive stress.^159^ Omega-3 fatty acids are emphasized in astronaut nutrition portfolios for immunomodulation and may complement reproductive redox support.^39^ Vitamin D requires routine mission supplementation because endogenous synthesis is absent in spacecraft; sufficiency supports musculoskeletal and immune axes relevant to reproductive fitness.^160^ Folate is essential for one-carbon metabolism and DNA methylation^161^; preconception/pregnancy data on folate status and methylation provide a rationale for monitoring and ensuring adequacy in space habitation scenarios.^162^ Melatonin improves oocyte mitochondrial function^163^ and reduces ROS^164^ in animal and human ART contexts,^165^ suggesting a candidate countermeasure to test in validated microgravity platforms. Given spaceflight-associated dysbiosis, personalized nutrition and space-tailored probiotics are being explored to help stabilize host-microbe dynamics.^166^ Prebiotic fibers and vetted probiotic strains may help maintain barrier integrity, immune tone, and metabolite production^40^; space-specific probiotic frameworks and “functional foods” are under active development.^41^

AG (short-radius, intermittent centrifugation), alone or combined with exercise, remains a leading integrated countermeasure for multi-system deconditioning and merits reproductive endpoints in future trials.^34^ AG platforms enable within-mission, gravity-controlled experiments to separate microgravity effects from other flight stressors. Inflight AG platforms, such as JAXA’s Mouse Habitat Unit and MARS platforms, have shown that artificial 1 g preserves musculoskeletal and gene expression profiles in rodents,^35^^,^^36^ providing a viable model for reproductive assessments. Recent missions such as MHU-5 and MHU-8 have investigated the effects of partial gravity on organ systems including the skeletal, immune, salivary gland, and ocular tissues.^37^^,^^38^^,^^167^ While these studies do not directly assess germ-cell development, they provide evidence supporting the feasibility of using AG platforms for organ-specific research and lay important groundwork for incorporating reproductive endpoints in future investigations.

Emerging technologies, such as artificial intelligence (AI), offer additional support for astronaut health by aiding in decision-making, data analysis, and outcome prediction.^42^ AI-powered risk prediction models, utilizing data from simulated environments, could be valuable for monitoring astronaut health and predicting risks of various conditions, including radiation-induced cancer and other health challenges encountered during space travel. Integrating AI with wearables can also support real-time physiological monitoring and on-orbit clinical decision support during extended missions.^168^

Cryopreservation and gene-editing techniques, such as gene silencing, also show potential for preserving reproductive health. Cryopreservation, through either slow freezing or vitrification, can preserve male fertility before spaceflight, safeguarding against the adverse effects of microgravity.^84^ However, cryopreservation can induce ROS injury to gametes and embryos; both sperm and oocytes exhibit cryo-damage linked to oxidative stress,^169^^,^^170^ with quality gains when antioxidants are incorporated into freezing/warming media.^171^^,^^172^ The choice of technique matters because vitrification generally minimizes ice-crystal formation and may reduce some injury relative to older slow-freezing methods, although optimized slow-freeze/rehydration protocols can perform competitively in some series.^173^ Given on-orbit radiation/thermal constraints, freeze-dried (lyophilized) sperm represents a practical alternative to liquid-nitrogen storage: mouse studies show healthy offspring from freeze-dried sperm held on the ISS for 9 months to nearly 6 years,^174^^,^^175^ and recent work has reported germline use of cryopreserved spermatogonial stem cells stored in space.^176^ These approaches reduce mass/cold-chain demands and, combined with antioxidant protocols (e.g., melatonin,^177^ NAC,^177^ mitochondria-targeted antioxidants^178^), may better align with exploration logistics.

Gene silencing, using RNA interference, holds promise for addressing microgravity-related gene expression changes that impact reproductive function. Clinically, RNA interference is delivered using lipid-nanoparticle (LNP) siRNA drugs (e.g., the patisiran class), including newer room-temperature-shippable formulations suitable for systemic dosing^179^; alternative platforms include adeno-associated virus (AAV) vectors that express shRNA/miRNA cassettes for sustained knockdown (used cautiously given insertional genotoxicity and immunogenicity risks),^180^ as well as engineered EVs/exosomes for tissue-directed delivery.^181^^,^^182^ Operationally, feasible routes could include periodic intravenous or intramuscular depot dosing,^183^^,^^184^ intravaginal/intrauterine gels for local female-tract delivery,^185^ or ultrasound-guided gonadal injections during scheduled medical operations.^186^^,^^187^ Nonetheless, the development of these approaches must also account for gender-specific reproductive challenges. For instance, long-term reductions in oxytocin levels and alterations in the hypothalamic-pituitary-adrenal axis observed post-spaceflight highlight the need for gender-specific countermeasures that can sustain reproductive and endocrine stability during prolonged missions.^9^

To address multi-generational concerns, mammalian models are essential for studying the effects of microgravity on reproductive physiology across generations, a key component for potential space colonization.^5^ Although animal data are not directly translational to humans, they remain indispensable for mechanism discovery, dose-finding, and multigenerational outcomes that cannot ethically be obtained in astronauts; these should be complemented by human organoids,^188^ iPSC-derived gametogenesis,^189^ and organ-on-chip systems^190^ to bridge species gaps. Furthermore, feasible countermeasures include pharmacological agents to mitigate oxidative stress and strategies to reduce radiation-induced DNA damage, both of which directly protect reproductive cell function.^16^^,^^84^ By contrast, prioritizing “telomere preservation” as a universal goal is less appropriate, since most astronaut telomere data derive from somatic cells, whereas germ cells and early embryos exhibit distinct telomere regulation through telomerase and recombination.^108^^,^^109^^,^^112^ Effective interventions should therefore emphasize minimizing oxidative and radiation stress while incorporating germline-specific monitoring of telomere and telomerase dynamics before targeted approaches are considered.

To sum up, future research should adopt an integrated approach to counter the combined effects of microgravity, radiation, and other space-specific environmental factors. Long-term studies on reproductive health in microgravity, coupled with advanced monitoring and intervention technologies, are likely to be vital for enabling sustainable human presence beyond Earth.^15^ Progress in biomarker discovery, such as spaceflight-associated miRNAs, offers promising avenues for targeted interventions; for example, modulation with antagomirs could help mitigate spaceflight-related gene dysregulation.^191^ Building on these insights, a pragmatic countermeasure framework would include: (i) platform-level approaches such as AG combined with structured exercise, (ii) safeguarded germline banking using optimized methods like vitrification or freeze-drying supplemented with antioxidant protection, and (iii) targeted, route-specific delivery of RNAi or biologic therapies. These measures, supported by animal multigeneration studies and human organoid models, together provide a coherent path toward protecting reproductive health while preserving crew safety and mission resources.

## Conclusion

### Summary of findings

This review highlights the significant effects of microgravity on reproductive cells, focusing on key physiological disruptions in both male and female reproductive systems. The findings emphasize that microgravity adversely affects gametogenesis, fertilization, and embryonic development through various mechanisms, including oxidative stress, DNA damage, and impaired cell signaling. Specific challenges such as reduced sperm motility, altered oocyte quality, and compromised early embryonic development are noted. Furthermore, EVs are found to be important mediators in supporting reproductive health, whose functions are significantly affected under microgravity conditions.

### Future research

The complex effects of microgravity on stem cell behavior, including impaired DNA repair and altered differentiation capacity, underscore the importance of understanding these underlying mechanisms. Such knowledge is essential for developing strategies to counteract adverse outcomes and to support reproductive health and development in space environments.

Nonetheless, current research faces several limitations. Most available data derive from animal models or non-germ somatic cell types, with direct studies on human gametes or embryos under true microgravity conditions remaining scarce. SMG platforms—such as clinostats or random positioning machines—provide valuable insights but may not fully replicate spaceflight stressors. Furthermore, cross-species differences in reproductive physiology complicate the translation of rodent or invertebrate findings to humans, necessitating caution when extrapolating results.

Because reproductive integrity underpins crew readiness and species continuity—and yields direct dividends for terrestrial reproductive medicine—future work must systematically address germline-relevant endpoints and translational pipelines. To this end, priorities include: (i) standardized, germline-relevant endpoints across validated platforms (ISS AG arms/partial-g, RPM/clinostat, and head-down bed rest with/without AG)—including endocrine panels, gamete/oocyte quality, cumulus-oocyte complex integrity, embryo lineage allocation, DNA damage/repair, mitochondrial function, EV cargo, and germline-specific telomere/telomerase assays, with results cross-validated across platforms (ISS, artificial-gravity centrifuges, and ground SMG) to separate gravity effects from confounders^192^^,^^193^; (ii) countermeasure testing that pairs AG with complementary approaches (e.g., antioxidant and radioprotective regimens, exercise, shielding) using dose-response designs relevant to lunar/Martian g-levels; and (iii) translational pipelines—human organoids, iPSC-derived gametogenesis, and organ-on-chip systems—to bridge species gaps and de-risk clinical considerations. In parallel, longitudinal biobanking, harmonized data standards, and cross-mission registries should be established to enable reproducible, meta-analytic evaluation of reproductive risks and countermeasure efficacy.

Integrative multi-omics are additionally recommended to resolve microgravity mechanisms in germ cells and early embryos, including single-cell and spatial transcriptomics, genome-wide DNA methylation and hydroxymethylation using bisulfite or oxBS-seq, histone-mark profiling such as H3K27me3 and H3K9me3 through ChIP-seq, small RNAs including miRNA and piRNA, EV cargo such as proteins and miRNAs, and metabolomics with isotope tracing. These datasets should be standardized and shared through open space-omics resources (e.g., SOMA^194^ and NASA GeneLab^195^), enabling cross-study synthesis and biomarker discovery. Methodologically, single-cell dual-omics in oocytes, including simultaneous transcriptome-proteome profiling^196^^,^^197^ and transcriptome-translatome sequencing,^197^ provide high-resolution readouts tailor-made for reproductive tissues. In addition, spatial omics approaches^198^ further enhance resolution and context in reproductive biology. Ultimately, these research efforts are expected to play a pivotal role in enabling safe and sustainable human reproductive health in space, supporting the long-term objectives of human space exploration and colonization.

### Limitations of the study

This systematic review has several limitations that should be acknowledged. First, much of the available evidence on the effects of microgravity on reproductive systems is derived from animal models or non-germline somatic cells, while direct investigations involving human gametes or embryos under true microgravity conditions remain limited. Second, many studies rely on simulated microgravity platforms, such as clinostats or random positioning machines, which, although informative, may not fully recapitulate the complex physiological stressors encountered during spaceflight. Third, substantial heterogeneity exists across studies with respect to species, experimental models, exposure durations, and measured endpoints, complicating cross-study comparisons and limiting the generalizability of conclusions. Finally, cross-species differences in reproductive physiology pose challenges for translating findings from rodent or invertebrate models to human reproductive health. These constraints highlight the need for more standardized, germline-relevant approaches and integrative strategies in future research.

## Acknowledgments

This work was supported by the 10.13039/100020595National Science and Technology Council of Taiwan (grant 113-2314-B-008-001) and Center for Astronautical Physics and Engineering from the Featured Area Research Center program within the framework of the Higher Education Sprout Project by the Ministry of Education in Taiwan.

## Author contributions

Y.-C.H. conceptualized the review article, provided guidance on the title and outline, and served as the corresponding author. C.-F.L., C.-Y.C., P.-T.L., and Y.-C.H. wrote the main body of the text, including the introduction, main sections, and conclusion, and performed the final revision of the manuscript. H.-H.L. created and provided the summarized tables and figures. All authors read and approved the final manuscript.

## Declaration of interests

All authors declare no financial or non-financial competing interests.

## Declaration of generative AI and AI-assisted technologies in the writing process

During the preparation of this manuscript, generative AI tools were used to assist with table organization and to improve sentence clarity and language flow. After using these tools, the authors reviewed and edited the content as needed and take full responsibility for the accuracy, integrity, and originality of the work.
